# Development of acceptable quality radiation dose levels for common computed tomography examinations: A focused multicenter study in United Arab Emirates

**DOI:** 10.3389/fpubh.2022.964104

**Published:** 2022-09-23

**Authors:** Wiam Elshami, Mohamed Abuzaid, Dlama Z. Joseph, H. O. Tekin, Hatem Ghonim

**Affiliations:** ^1^Department of Medical Diagnostic Imaging, College of Health Sciences, University of Sharjah, Sharjah, United Arab Emirates; ^2^Department of Radiology, Abubakar Tafawa Balewa University Teaching Hospital, Bauchi, Nigeria; ^3^Computer Engineering Department, Faculty of Engineering and Natural Sciences, Istinye University, Istanbul, Turkey; ^4^College of Medicine, University of Sharjah, Sharjah, United Arab Emirates

**Keywords:** Diagnostic reference level (DRL), Acceptable Quality Dose (AQD), Computed Tomography (CT), radiation dose, radiation protection, radiation safety, patient safety

## Abstract

**Purpose:**

Diagnostic Reference Level (DRL) is a practical tool for radiation dose optimization, yet it does not indicate the patient size or image quality. The Acceptable Quality Dose (AQD) introduced to address the limitations of the DRLs and it is based on image quality, radiation dose, and patient weight. The aim of this study is to establish the AQD for adult patients' undergoing Computed Tomography (CT) examinations (Head, chest, abdomen).

**Methods:**

This study is conducted in the four main hospitals at the Ministry of Health and Prevention. Patient information and exposure parameters were extracted. All the acceptable images are scored for their quality assessments. Data is classified as seven weight groups, <50, 50–59, 60–69, 70–79, 80–89, 90–99, and ≥100 kg. The mean ± SD, median, and 75th are calculated for the CTDIvol and DLP for each weight group per examination.

**Results:**

Out of 392, 358 CT examinations are scored with acceptable quality. The median CTDIvol values for the weight groups are obtained as 24.6, 25.4, 25.4, 25.0, 26.0, 27.0, and 29.0 mGy. Moreover, median DLP values are obtained as 576.7, 601.0, 616.5, 636.1, 654.0, 650.0, 780.0, and 622.5 mGy.cm, respectively, for head CT without Contrast Media (CM). Similar calculation for head CT with (CM), chest without CM, abdomen without CM, and chest and abdomen (with and without CM) CTs are presented.

**Conclusion:**

Images with bad, unacceptable and higher than necessary qualities contribute to increasing patient dose and increasing the DRLs. The AQD for the selected examinations were lower than the proposed DRLs in the United Arab Emirates. The integration of image quality and patients size in the assessment of the AQD values provide effective model to compare radiation dose indices within facility and compare with others. The obtained results may be useful in terms of improving dose and the diagnostic quality in the national and international levels.

## Introduction

Technology advancement increased the clinical applications of radiation in the medical field, and an increased absorbed radiation dose is expected for patients undergoing radiology examinations. Nevertheless, a higher radiation dose is reported for patients experiencing computed tomography (CT) examinations (1, 2). The primary role of the radiology team is to provide quality diagnostic information while also ensuring radiation safety. With the advent of CT in the early 1970's, novel technologies and processes have been frequently put into action to mitigate the radiation footprint and increase image quality (3). The ALARA (as low as reasonably achievable) principle had been instrumental in the balancing of stochastic risks and benefits associated with the use of ionizing radiation for decades. It aims to maintain exposures to ionizing radiation as low as possible while providing adequate diagnostic information and complying with regulatory requirements (4). Meanwhile, the term of dose optimization process is one of the key concepts of radiation protection in medical imaging procedures. It involves compromising between the image quality and dose to the patient; the dose should not be higher than necessary to achieve an image quality needed for diagnostic purposes (1).

Regarding patient benefits, it is well–known that Diagnostic reference level (DRL) is a useful dose optimization tool. First, it was introduced in 1996 by the International Commission on Radiological Protection. DRL value is a radiation dose quantity for a typical examination for standard-sized patients. DRL is specified by the equipment type or standard phantom, which is not expected to exceed (5). When the median value of the DRL quantity for a representative sample of standard-sized patients at a facility exceeds the local or national DRL value, a DRL is considered to have been exceeded. DRLs identify the diagnostic processes and diagnostic facilities that require further radiation dose adjustment to assure radiation safety.

Further, it also guides in optimizing radiation dose when the median dose for an examination exceeds a DRL at any given facility (5). In CT, DRL calculation is based on volume CT dose index (CTDI) and dose-length product (DLP) on the standard patient or phantom, and it is applied to all patients regardless of their body build (6). Patients with a big frame may not always be suitable candidates for DRL. They need a higher radiation dose than the DRL value in order to provide diagnostic images of appropriate quality. DRL is based on retrospective analysis of a facility and examination results, while optimization needs addressing the prospective scenario of optimizing care for the patient at hand. Moreover, the majority of the dose surveys conducted for DRL considers acceptable image quality rather than confirming and measuring the individual image quality itself (7). DRL has played a useful role in optimization, but DRL is based on radiation dose only. Therefore, it does not indicate image quality or patient size (8). The relationship between image noise, image quality and patient size is well–documented (9). Indeed, this necessitates radiation dose optimization based on patient size and diagnostic image quality (7).

The Acceptable Quality Dose (AQD) is a newly presented alternative concept that addresses the majority of DRL's drawbacks. Based on image quality, radiation dose, and patient weight, it is a bottom-up optimization strategy (10). Modern CT systems change the milliampere (tube current) during each rotation and adjust the exposure based on the patient's size to attain certain picture noise levels. Therefore, manual selection may be required for extremely tiny or very big patients to get high-quality images (7). However, AQD suggests dividing adult patients into 10 kg and children into 5 Kg intervals to accommodate the body habitus (7). The radiologist evaluates image quality first, and dose indices are only calculated for images found to be of acceptable quality. The scoring method may be used to evaluate image quality, and there was a strong connection between objective and subjective image quality evaluations (7). The primary objective of this research is to determine the AQD arising from different CT examination techniques for adult patients in four United Arab Emirates hospitals. Considering the importance of dose reduction strategies for public health, the obtained results may be useful in terms of improving dose and the diagnostic quality in the national and international levels.

## Materials and methods

The data for adult patients was collected from four CT scanners from four hospitals under the Ministry of Health and Prevention (MOHAP). The most common CT examinations performed for patients aged 19 years and above were included in the study. These were the CT of the head (with and without Contrast Media (CM), chest without CM, abdomen without CM, and chest and abdomen (with and without CM). Data was collected over a period of 3 months, 10 to 20 consecutive patients included in the study. Patient information and exposure parameters were extracted, including patient age, sex, weight, height, date of examination, and dose indexes CTDIvol (mGy) and DLP, were collected from the console of each scanner.

### Image quality assessment (non-objective evaluation)

All images were assessed for image quality according to scoring criteria ranging from 1 to 4. A score of 1 represents bad image quality (required features/anatomy not seen), a score of 2 represents an unacceptable image quality (images where diagnostic interpretation cannot be done), score of 3 represents an acceptable quality (images with adequate information for diagnostic interpretation), and score of 4 represents quality higher than necessary (image quality much better than that is needed for diagnostic interpretation). Images were evaluated by three radiologists. To assess the AQD, only images with acceptable quality (score of 3) were included in the study and images with a score of 1, 2, and 4 were excluded. CT procedures with an acceptable score of image quality (score 3) were classified into head [with and without Contrast Media (CM)], chest without CM, abdomen without CM, and chest and abdomen (with and without CM). Then, data grouped into seven weight groups, <50, 50–59, 60–69, 70–79, 80–89, 90–99, and ≥100 Kg.

### Data analysis

Descriptive statistical analysis was performed with IBM SPSS Statistics version 22. The mean ± SD, median (50th percentile), and third quartile (75th percentile) were calculated for the CTDIvol and DLP. Additionally, the averages of the age of patients in each weight group per examination, the total CTDIvol, and total DLP per examination were calculated. Finally, the median and 75^th^% for the DLP and CTDI_vol_ were compared with the United Arab Emirates DRL (11).

## Results

A total of 392 CT procedures were collected, and 358 CT procedures were deemed of acceptable quality (score of 3) were included in the study. It was found that 34 (9.5%) CT procedures were not scored as 3; (4.2%, *n* = 15), (3.6%, *n* = 13), and (1.7%, *n* = 6) were score 4, score 2, and score 1, respectively. nine (7.3%) and 3 (0.8%) CT exams were scored 1 and 2, respectively. The majority of the CT images belonged to patients who weighed between 70–79kg, accounting for 29.3% of the total. The frequency of total scans according to weight categories and classification of CT examination (Table 1). Head CT without CM (*n* = 79) composed the largest component, which is 22.1%, followed by CT chest (*n* = 68), which was 19%. Tables 2–**7** present the data for CT head without and with CM, chest without CM, abdomen without CM, and chest and abdomen without and with CM, respectively, along with their mean, third quartile and median values. The median values for each examination is considered as their respective AQD. Table 2 shows the distribution of Head CT without CM images in patients between the weight groups of <51, 51–60, 61–70, 71–80, 81–90, 91–100, and more than 100 kg was 2.5, 11.4, 20.3, 26.6, 26.6, 8.9, and 3.8%, respectively. The median CTDIvol for these CT were 24.6, 25.4, 25.4, 25.0, 26.0, 27.0, and 29.0 mGy, respectively. The median DLP values of these examinations were 576.7, 601.0, 616.5, 636.1, 654.0, 650.0, and 780.0 mGy cm, respectively.

**Table 1:** Frequency of weight groups.

**Weight groups**	**Head** **CT without CM**	**Head CT** **with contrast**	**Chest CT without** **CM**	**Abdomen and pelvis** **without CM**	**Abdomen and pelvis** **with CM**	**Abdomen without** **CM**	**Total**
<50 kg	2.5% (*n* = 2)	1.5% (*n* = 1)	5.9% (*n* = 4)	5.3% (*n* = 2)	9.3% (*n* = 4)	6.2% (*n* = 4)	4.7%(*n* = 17)
50–59 kg	11.4% (*n* = 9)	9.2% (*n* = 6)	5.9% (*n* = 4)	26.3% (*n* = 10)	7.0% (*n* = 3)	10.8% (*n* = 7)	10.9% (*n* = 39)
60–69 kg	20.3% (*n* = 16)	20.0% (*n* = 13)	30.9% (*n* = 21)	21.1% (*n* = 8)	27.9% (*n* = 12)	21.5% (*n* = 14)	23.5% (*n* = 84)
70–79 kg	26.6% (*n* = 21)	36.9% (*n* = 24)	29.4% (*n* = 20)	26.3% (*n* = 10)	27.9% (*n* = 12)	27.7% (*n* = 18)	29.3% (*n* = 105)
80–89 kg	26.6% (*n* = 21)	18.5% (*n* = 12)	20.6% (*n* = 14)	15.8% (*n* = 6)	11.6% (*n* = 5)	21.5% (*n* = 14)	20.1% (*n* = 72)
90–99 kg	8.9% (*n* = 7)	13.8% (*n* = 9)	5.9% (*n* = 4)	5.3% (*n* = 2)	7.0% (*n* = 3)	12.3% (*n* = 8)	9.2% (*n* = 33)
≥100 kg	3.8% (*n* = 3)	0% (*n* = 0)	1.5% (*n* = 1)	0% (*n* = 0)	9.3% (*n* = 4)	0% (*n* = 0)	2.2% (*n* = 8)
Total	22.1% (*n* = 79)	18.2% (*n* = 65)	19.0% (*n* = 68)	10.6% (*n* = 38)	12.0% (*n* = 43)	18.2% (*n* = 65)	100% (*n* = 358)

**Table 2:** Distribution of head CT without CM for patients in different weight groups.

**Weight group**		**<50 kg**	**50–59 kg**	**60–69 kg**	**70–79 kg**	**80–89 kg**	**90–99 kg**	**≥100 kg**	**Total**
No. of patients		2	9	16	21	21	7	3	79
Mean weight		47.0	57.0	102.7	107.0	86.0	96.1	112.0	74.9
Mean age		23.5	62.3	40.4	47.2	48.5	51.3	108.5	43.9
CTDI_vol_	Median	24.6	25.4	25.4	25.0	26.0	27.0	29.0	25.4
	75%	24.9	31.6	32.5	33.26	34.2	35.6	37.1	34.1
	Mean	24.6	25.6	26.9	27.6	28.1	29.2	32.3	29.7
	SD	1.2	6.3	10.6	7.5	6.0	4.3	1.5	7.9
DLP	Median	576.7	601.0	616.5	636.1	654.0	650.0	780.0	622.5
	75%	598.1	605.8	616.5	690.8	719.6	729.0	799.2	695.2
	Mean	506.7	511.4	521.4	556.3	554.6	674.6	806.6	555.9
	SD	60.5	374.9	124.0	311.6	338.1	242.2	57.5	240.4

Table 3 shows the distribution of Head CT with CM images in patients between the weight groups of 51–60, 61–70, 71–80, 81–90, and 91–100 kg was 9.2, 20.0, 36.9, 18.5, and 13.8%, respectively. The median CTDIvol for these CT were 33.6, 34.7, 34.8, 35.3, and 37.0 mGy, respectively. The median DLP values of these examinations were 407.5, 527.7, 585.4, 607.4, and 902.1 mGy cm, respectively. Table 4 shows the distribution of chest CT without CM images in patients between the weight groups of <51, 51–60, 61–70, 71–80, 81–90, and 91–100 kg was 5.9, 5.9, 30.9, 29.4, 20.6, 5.9, and 1.5%, respectively. The median CTDIvol for these CT were 4.5, 4.7, 6.4, 6.6, 10.6, and 10.5 mGy, respectively. The median DLP values of these examinations were 145.0, 162.5, 196.5, 207.2, 275.0, and 326.0 mGy cm, respectively.

**Table 3:** Distribution of head CT with CM for patients in different weight groups.

**Weight group**		**<50 kg**	**50–59 kg**	**60–69 kg**	**70–79 kg**	**80–89 kg**	**90–99 kg**	**≥100 kg**	**Total**
No. of patients		1.0	6.0	13.0	24.0	12.0	9.0	0.0	65.0
Mean weight			59.3	66.4	77.5	86.1	94.7		78.5
Mean age			33.7	28.4	43.6	40.2	60.6		41.8
CTDI_vol_	Median		33.6	34.7	34.8	35.3	37.0		35.6
	75%		35.6	37.9	38.85	39	45.3		38.9
	Mean		30.7	37.5	41.3	37.3	41.1		38.6
	SD		12.0	10.7	44.2	21.2	11.3		29.0
DLP	Median		407.5	527.7	585.4	607.4	902.1		576.9
	75%		545.2	576.9	764.9	811.7	995.1		798.9
	Mean		482.2	593.3	607.1	679.6	709.3		619.9
	SD		250.2	337.5	303.8	318.8	395.6		316.6

**Table 4:** Distribution of chest CT without CM for patients in different weight groups.

**Weight group**		**<50 kg**	**50–59 kg**	**60–69 kg**	**70–79 kg**	**80–89 kg**	**90–99 kg**	**≥100 kg**	**Total**
No. of patients		4.0	4.0	21.0	20.0	14.0	4.0	1.0	68.0
Mean weight		47.0	129.2	150.0	75.7	86.5	148.5		74.0
Mean age		82.3	88.0	44.3	101.5	56.6	119.6		49.6
CTDI_vol_	Median	4.5	4.7	6.4	6.6	10.6	10.5		5.7
	75%	5.1	4.8	6.0	10.2	11.1	13.2		9.7
	Mean	4.3	4.4	5.3	8.3	9.5	11.2		7.2
	SD	1.3	0.6	2.1	5.1	11.7	4.2		4.1
DLP	Median	145.0	162.5	196.5	207.2	275.0	326.0		200.0
	75%	163.0	182.1	239.0	275.2	358.3	344.9		275.2
	Mean	118.6	176.0	200.2	237.6	266.9	270.8		225.3
	SD	75.1	36.0	49.3	139.7	104.0	58.7		101.7

Table 5 shows the distribution of abdomen CT without CM images in patients between the weight groups of <51, 51–60, 61–70, 71–80, 81–90, and 91–100kg was 6.2, 10.8, 21.5, 27.7, 21.5, and 12.3%, respectively. The median CTDIvol for these CT were 3.5, 4.7, 5.6, 7.0, 7.8, and 8.5 mGy, respectively. The median DLP values of these examinations were 493.3, 512.0, 530.5, 588.0, 620.9, and 642.9 mGy cm, respectively. Table 6 shows the distribution of CT abdomen and pelvis without CM images in patients between the weight groups of <51, 51–60, 61–70, 71–80, 81–90, 91–100, and >100 Kg was 9.3, 7.0, 27.9, 27.9, 11.6, 7.0, and 9.3%, respectively. The median CTDIvol for these CT were 4.6, 5.3, 7.0, 8.3, 8.8, 9.4, and 11.5 mGy, respectively. The median DLP values of these examinations were 505.2, 561.4, 601.9, 635.5, 656.1, 693.9, and 713.3 mGy cm, respectively. Table 7 shows the distribution of CT abdomen and pelvis without CM images in patients between the weight groups of <51, 51–60, 61–70, 71–80, 81–90, and 91–100 kg was 5.3, 26.3, 21.1, 26.3, 15.8, and 5.3%, respectively. The median CTDIvol for these CT were 8.2, 9.0, 10.5, 10.9, 11.2, and 11.8 mGy, respectively. The median DLP values of these examinations were 437.4, 490.0, 593.5, 624.5, 696.9, and 739.4 mGy cm, respectively.

**Table 5:** Distribution of abdomen without CM CT for patients in different weight groups.

**Weight group**		**<50 kg**	**50–59 kg**	**60–69 kg**	**70–79 kg**	**80–89 kg**	**90–99 kg**	**≥100 kg**	**Total**
No. of patients		4	7.0	14.0	18.0	14.0	8.0		65.0
Mean weight		47.0	54.9	67.0	76.7	85.2	94.3		74.1
Mean age		25.0	56.7	38.8	76.7	42.2	49.0		42.7
CTDI_vol_	Median	3.5	4.7	5.6	7.0	7.8	8.5		7.7
	75%	4.25	4.845	5.3	6.5575	7.4975	9.055		11.9
	Mean	4.5	4.9	5.9	6.6	9.9	10.2		16.6
	SD	3.9	2.2	3.9	3.4	2.7	5.8		11.1
DLP	Median	493.3	512.0	530.5	588.0	620.9	642.9		746.8
	75%	500.125	520.95	602.5	660.325	759.925	788.25		1596.7
	Mean	493.3	527.2	645.8	721.7	730.7	784.2		1243.1
	SD	19.4	159.7	280.0	288.4	144.9	121.8		1070.8

**Table 6:** Distribution of abdomen and pelvis without contrast CT for patients in different weight groups.

**Weight group**		**<50 kg**	**50–59 kg**	**60–69 kg**	**70–79 kg**	**80–89 kg**	**90–99 kg**	**≥100 kg**	**Total**
No. of patients		4.0	3.0	12.0	12.0	5.0	3.0	4.0	43.0
Mean weight		49.0	57.3	66.9	75.4	87.0	94.7	109.0	74.7
Mean age		40.0	47.3	36.7	47.0	41.8	45.0	36.5	41.4
CTDI _vol_	Median	4.6	5.3	7.0	8.3	8.8	9.4	11.5	8.7
	75%	4.9	6.2775	7.905	9.0725	11.0775	12	12.25	11.3
	Mean	4.6	5.3	6.4	7.8	10.6	9.9	11.8	9.2
	SD	0.8	2.8	2.8	2.1	4.7	2.4	1.0	3.7
DLP	Median	505.2	561.4	601.9	635.5	656.1	693.9	713.3	589.0
	75%	530.875	615.1975	740.4	756.05	802.63	817.74	836.15	802.6
	Mean	505.2	561.4	644.8	671.5	749.8	746.1	834.1	726.1
	SD	72.8	152.3	215.8	217.9	209.3	161.4	142.8	207.5

**Table 7:** Distribution of abdomen and pelvis with CM CT for patients in different weight groups.

**Weight group**		**<50 kg**	**50–59 kg**	**60–69 kg**	**70–79 kg**	**80–89 kg**	**90–99 kg**	**≥100 kg**	**Total**
No. of patients		2.0	10.0	8.0	10.0	6.0	2.0		38.0
Mean weight		50.0	58.2	64.3	77.2	88.0	96.0		70.7
Mean age		19.0	47.4	32.5	33.3	29.3	34.0		35.2
CTDI _vol_	Median	8.2	9.0	10.5	10.9	11.2	11.8		11.0
	75%	8.575	11.9	12.5	12.85	12.05	12.05		12.0
	Mean	7.2	9.7	10.3	11.4	11.3	11.8		9.7
	SD	1.2	2.5	3.1	1.9	1.0	0.7		2.3
DLP	Median	437.4	490.0	593.5	624.5	696.9	739.4		650.0
	75%	463.7	616.9	638.75	650	696.9	760.65		696.9
	Mean	437.4	544.5	613.5	546.8	671.4	739.4		573.1
	SD	74.4	150.3	330.5	141.7	358.5	60.1		169.3

## Discussion

In radiology, the DRL is one of the approaches for optimizing radiation dosage. However, the United Arab Emirates is now working intensively and diligently to build a national CT DRL that can be adopted nationwide. These DRL were given to the IAEA Expert Mission to the United Arab Emirates in April 2015 as part of the UAE-IAEA technical cooperation program for the 2014–2015 cycle. The DRLs listed have been approved as the United Arab Emirates's first diagnostic reference values in 2018. Based on the recommendations of the IAEA Expert Mission, two assumptions were made that the picture quality was satisfactory and that all equipment were suitable for clinical use. Further studies conducted after 2015 as a part of this effort by (12, 13). The results of the current study demonstrate the first initiative providing AQDs for CT patients. While the DRL provide estimation of the radiation dose, the AQD approach based on image quality, radiation dose, and patient's weight DRL does not account patient weight (12). The results of the current study showed that 9.5% CT procedures were not within the acceptable image quality. Including images with unacceptable quality will impact the dose assessment and the radiation dose indices will not be accurate. It worth mentioning that 4.2% of the images were deemed to have quality higher than necessary, which infer increasing radiation dose. The purpose of dose optimization is to achieve the lowest dose necessary to answer clinical issues. The AQD is optimization approach addressing the limitations of DRL including image quality and patient's weight (12).

The patient weight parameter was introduced specifically during data collection rather than presuming that all populations are of typical size. Individuals weighing 60 kg are considered average in African and Asian countries, and 70 kg in affluent ones. Despite being an Asian country, the United Arab Emirates ranks fifth globally for obesity. The study demonstrates that 20.1% of patients weighed between 80–89 kg and 11.4% were above 90 Kg. DRLs are set for a standard-sized patient (70 kg ± 15 kg), but individual sizes vary in different countries. The weight of the patients in the previous study ranged from 40.0 to 123.0 kg, with medians between 76.0 and 80.0 kg for various assessments. Studies in the current context showed a high prevalence of overweight (43.0%) and obesity (32.3%) in the United Arab Emirates (13). Table 8 shows the AQD, results of the current study, and DRL for CTDIvol and total DLP per examination, including the median and 75% percentile. Table 8 compares the study results with the previous published DRLs in UAE. The initial United Arab Emirates DRL reports lack the CTDI_vol_ data. The current results were lower than the previous 75^th^ proposed DRLs. The reported CDTI_vol_ and DLP in the current study are lower in comparison with the value reported in the literature (14, 15). This can be due to the fact the CDTI and DLP measured here are for the images with acceptable quality (score of 3) were included in the study and images with a score of 1, 2, and 4 were excluded. It is being discussed by Rehani (2015) that the acceptable quality dose can be obtained at doses <25% of the national dose distribution (7).

**Table 8:** Results of the current study compared to the national DRL.

	**CTDI**_**vol**_						**DLP**					
	**AQD**		**UAE (2018)**		**UAE 2020**		**AQD**		**UAE (2018)**		**UAE 2020**	
	**Median**	**75th%**	**Median**	**75th%**	**Median**	**75th%**	**Median**	**75th%**	**Median**	**75th%**	**Median**	**75th%**
Head CT without CM	25.4	34.1	-	-	29.9	39.6	622.5	695.2	-	871	639.3	693.1
Head CT with Contrast	35.6	38.9	-	-	41.4	48	576.9	798.9	-	1,071	772.2	818.7
Chest CT without CM	5.7	9.7	-	-	7.1	10.2	200	275.2	-	443	251.7	276.2
Abdomen without CM	7.7	11.9	-	-	NA	NA	746.8	1,596.7	-	671	NA	NA
Abdomen and pelvis without CM	8.7	11.3	-	-	11	13.5	589	802.6	-	-	656.2	811.8
Abdomen and pelvis with CM	11	12	-	-	16.9	20.4	650	696.9	-	-	606.5	1,023.1

The implementation of the proposed DRLs and standardized protocols could be the justification for the relatively lower dose in this study. Furthermore, due to the comparable technology and procedure employed, the relatively new scanners were made by a single vendor (GE), resulting in significant homogeneity in the radiation outputs. In general, protocol variations can change the radiation dose; thus, the same scanner may provide greater or lower doses than the DRLs. The patient dose was positively affected by practice of following a standard protocol for all patients. Patients' radiation doses can be reduced by altering protocols and exposure variables in accordance with their size and weight. Such opportunities can be addressed by training and continuing education programs, which can boost technologists' confidence. The current study with conjunction of available DRLs may be used to monitor the patient dose and as a baseline for the current procedures. Future studies based on the indication and clinical reasons are recommended as it is expected to have different CTDI and DLP for each indication. Similarly, follow up cases can be investigated.

## Data availability statement

The original contributions presented in the study are included in the article/supplementary material, further inquiries can be directed to the corresponding author.

## Ethics statement

The studies involving human participants were reviewed and approved by MOHAP Research Ethics Committee (reference number MOHAP/REC-18/2018). Written informed consent was not required for this study and waived due to retrospective data collection.

## Author contributions

WE, MA, and DJ: were responsible for conceptualization, writing and original draft preparation. MA and WE developed the methodology. HT, WE, and DJ performed formal analysis. MA and HG assisted with the investigation. HT, MA, and WE: data curation. HT and HG: undertook writing, review and editing, were responsible for supervision. MA, HT, and DJ contributed to project administration. All authors have read and agreed to the published version of the manuscript.

## Funding

The project is funded by the University of Sharjah, Research Institute for Medical and Health Science (1901050246).

## Conflict of interest

The authors declare that the research was conducted in the absence of any commercial or financial relationships that could be construed as a potential conflict of interest.

## Publisher's note

All claims expressed in this article are solely those of the authors and do not necessarily represent those of their affiliated organizations, or those of the publisher, the editors and the reviewers. Any product that may be evaluated in this article, or claim that may be made by its manufacturer, is not guaranteed or endorsed by the publisher.
